# CO_2_ Reactivity but Not CO_2_‐Induced Orexin/c‐Fos Colocalization Differentially Predicts Alcohol‐Seeking Behaviour After Extinction and Retrieval‐Extinction in Rats

**DOI:** 10.1111/adb.70116

**Published:** 2026-01-22

**Authors:** Marissa Raskin, Marcelle Olvera, Kylee A. Smith, Roberto Cofresí, Jason Shumake, Michael J. Telch, Michael W. Otto, Jasper A. J. Smits, Rueben Gonzales, Hongjoo J. Lee, Marie‐H. Monfils

**Affiliations:** ^1^ Interdisciplinary Neuroscience Program University of Texas at Austin Austin Texas USA; ^2^ Department of Psychology University of Texas at Austin Austin Texas USA; ^3^ Department of Psychological Sciences University of Missouri Columbia Missouri USA; ^4^ Department of Psychological & Brain Sciences Boston University Boston Massachusetts USA; ^5^ College of Pharmacy University of Texas at Austin Austin Texas USA

**Keywords:** extinction, individual differences, orexin, Pavlovian conditioning

## Abstract

Cues associated with alcohol consumption can trigger cravings, seeking behaviour and relapse after abstinence in individuals with alcohol use disorder (AUD). These conditioned responses can be attenuated through extinction learning, a core component of cue exposure therapy (CET). CET is effective in some individuals with AUD but not all, so it is necessary to develop strategies to identify and intervene with individuals unlikely to benefit from CET. Another method for attenuating conditioned responding is retrieval‐extinction, which renders the original associative memory labile via distinct neural mechanisms. We recently demonstrated that CO_2_ reactivity predicts extinction memory for both fear and food cues, and fear memory after retrieval‐extinction, and CO_2_‐induced orexin/c‐Fos colocalization predicts fear extinction memory. The purpose of the current study was to examine whether the predictive power of CO_2_ reactivity might extend to alcohol‐seeking behaviour after extinction or retrieval‐extinction in male and female rats. We also examined the relationship between CO_2_ reactivity, return of alcohol‐seeking behaviour and CO_2_‐induced orexin/c‐Fos colocalization. Male and female rats first underwent alcohol drinking induction in the homecage followed by dependence via exposure to chronic intermittent ethanol vapour or control air and homecage drinking. All rats then underwent Pavlovian alcohol conditioning followed by either standard extinction or retrieval‐extinction. They then received a long‐term memory (LTM) test and CO_2_ challenge followed by euthanasia for brain harvesting. CO_2_ reactivity differentially predicted LTM after extinction and retrieval‐extinction. There were no relationships between orexin/c‐Fos colocalization and CO_2_ reactivity or LTM. The predictive power of CO_2_ reactivity extends to alcohol‐seeking behaviour after extinction and retrieval‐extinction in alcohol dependent and nondependent male and female rats, while its relationship with orexin/c‐Fos colocalization does not. CO_2_ reactivity could be used as a screening tool to determine whether an individual may be a good candidate for CET or a retrieval‐extinction–based approach.

## Graphical abstract

CO_2_ reactivity differentially predicted alcohol‐seeking behaviour in alcohol dependent and non‐dependent male and female rats after extinction and retrieval‐extinction. However, there were no relationships between orexin/c‐Fos colocalization and CO_2_ reactivity or return of alcohol‐seeking behaviour. This supports the potential of CO_2_ reactivity to be used as a screening tool to determine whether an individual may be a good candidate for cue exposure therapy or a retrieval‐extinction‐based approach.

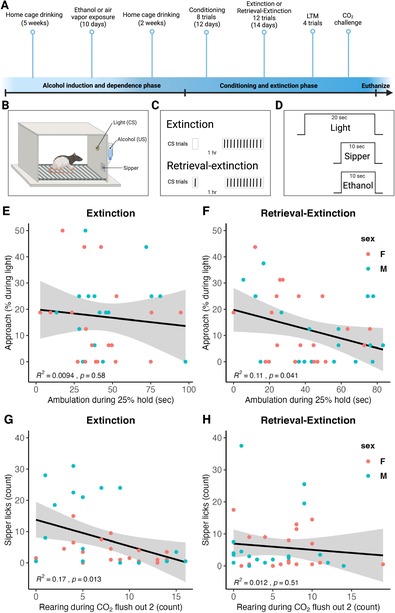

## 1 Introduction

Alcohol use disorder (AUD) is characterized by persistent conditioned responses to previously neutral stimuli—visual, olfactory and contextual cues (conditioned stimuli, CS) associated with alcohol consumption (unconditioned stimulus [US])—that can trigger cravings and even relapse after abstinence [1]. These responses can be attenuated through extinction training, where cues are repeatedly presented without the previously learned outcome of alcohol consumption. Cue exposure therapy (CET) is based on the principles of extinction training and is effective in reducing symptoms in some patients with AUD, but not all [2]. This is in part because extinction memories do not directly weaken the original CS‐US memory but instead form a new CS‐noUS memory that may temporarily inhibit conditioned responses, allowing conditioned alcohol‐seeking behaviour to return with the passage of time, stress or change in context, leading to relapse [3, 4]. As such, it is important to develop strategies to identify and intervene with individuals likely to fail CET.

One such strategy is to consider individual differences in extinction learning that could be used to identify likely responders to CET. Despite this, there has been little research on individual differences in alcohol extinction that could be used to identify which patients would be good candidates for CET, and which are likely to relapse and would thus be better suited for an alternative treatment. However, there has been extensive work to identify predictors of fear extinction memory [5]. There is overlap in the neural mechanisms underlying extinction of fear and reward cues (such as alcohol) [6, 7]. One overlapping mechanism is that of the orexin system, a neuropeptide synthesized in the lateral hypothalamus (LH) that is implicated in a variety of motivated behaviours [8, 9, 10]. In fear, increased activity of orexin neurons is associated with greater fear expression during extinction training, and orexin antagonism facilitates extinction training [11, 12]. In alcohol, increased orexin activity is associated with greater alcohol seeking during renewal and reinstatement [13, 14] and orexin antagonism prevents spontaneous recovery, renewal and reinstatement of alcohol seeking and consumption [14, 15, 16, 17, 18].

In rats, orexin neurons are also activated by exposure to carbon dioxide (CO_2_) [19, 20]. Our lab previously found that in rats, behavioural reactivity to a CO_2_ challenge negatively predicts fear extinction memory and orexin/c‐Fos colocalization, and the number of CO_2_‐activated orexin neurons positively predicts fear extinction memory [21]. We recently extended these findings to show that CO_2_ reactivity predicts extinction memory in food‐conditioned rats [22]. Thus, CO_2_ reactivity might be used as a proxy for orexin activity to predict which individuals respond well to extinction of fear and reward cues. This alternative assessment method is important, given that, in humans, orexin activity cannot be quantified in a reliable and noninvasive manner [23, 24].

Another strategy to prevent relapse is to directly weaken the original CS‐US memory instead of forming a CS‐noUS memory. This can be achieved through retrieval‐extinction, wherein presentation of an isolated CS (retrieval) renders the CS‐US memory labile such that it can then be updated with extinction training [25]. Originally found to prevent the return of fear in rats and later humans, retrieval‐extinction has since been found to attenuate conditioned responding to food, cocaine and alcohol in rats and morphine in rats and humans [26, 27, 28, 29, 30, 31, 32, 33]; see [34] for a meta‐analysis. We recently found that CO_2_ reactivity also positively predicts fear expression after retrieval‐extinction, albeit in a different manner and to a lesser degree [35]. Unlike extinction, the role of orexin in retrieval‐extinction has not been examined.

Here, we examined whether the predictive power of CO_2_ reactivity might extend to alcohol‐seeking behaviour after extinction or retrieval‐extinction in alcohol dependent and nondependent male and female rats. We also examined the relationship between CO_2_ reactivity, return of alcohol‐seeking behaviour and CO_2_‐induced orexin/c‐Fos colocalization.

## 2 Methods

### 2.1 Subjects and Experimental Timeline

**FIGURE 1:**
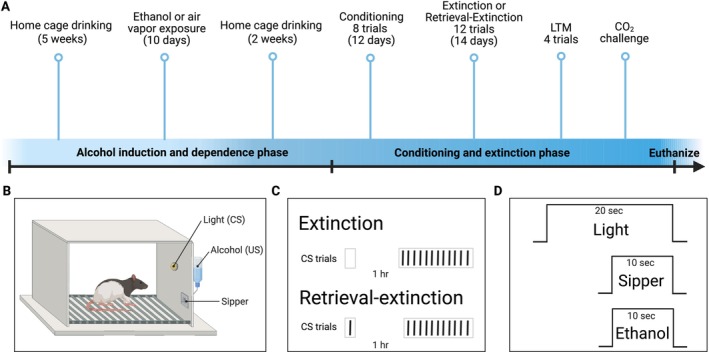
Experimental timeline (A) and details of conditioning procedures. Male (*n* = 34) and female (*n* = 42) rats first underwent alcohol drinking induction in the homecage followed by dependence via exposure to ethanol vapour (*n* = 34) or control air (*n* = 42) and homecage drinking. All rats then underwent alcohol conditioning with 8 trials of a light (CS) followed by a retractable sipper containing ethanol (US) (B, D) daily for 12 days. Next, rats were assigned to receive either standard extinction (*n* = 36) or retrieval‐extinction (*n* = 40) (C) with 12 trials of CS‐sipper pairings daily for 14 days. All rats then underwent a long‐term memory (LTM) test with 4 CS‐sipper pairings. They then received a CO_2_ challenge followed by euthanasia for brain harvesting. Figure created with http://BioRender.com.

The experimental timeline is summarized in Figure 1A. Subjects consisted of adult male (*n* = 34) and female (*n* = 42) Long‐Evans rats (Envigo, Indianapolis, IN, USA). Upon arrival, male rats weighed approximately 250–275 g and female rats weighed 220–225 g. Rats were singly housed in temperature‐ and humidity‐controlled transparent polyethylene cages with ad libitum access to food and water on a 12‐h light cycle with lights on at 7:00 AM. Rats first underwent alcohol drinking induction (and dependence in half the sample) over the course of 12 weeks. They then received 12 daily conditioning sessions of eight trials of a light cue paired with a retractable sipper containing ethanol (Figure 1B). Rats were then assigned to receive 14 daily sessions of either extinction or retrieval‐extinction consisting of 12 trials of light‐empty sipper pairings (Figure 1C). Two days after the final extinction session, they received a long‐term memory (LTM) test with four trials of light‐empty sipper pairings. All rats then received a CO_2_ challenge prior to euthanasia. Brains were harvested and processed for orexin‐cFos immunohistochemistry and those with a minimum of three bilateral sections suitable for cell counting were included in the analysis (*n* = 63). All procedures were conducted in compliance with the National Institutes of Health *Guide for the Care and Use of Experimental Animals* and were approved by the Institutional Animal Care and Use Committee of the University of Texas at Austin.

### 2.2 Alcohol Induction and Dependence

To induce alcohol drinking, rats received intermittent (MWF) access to 15% unsweetened ethanol solution diluted with tap water (v/v [15E]; 95% Pharmco‐AAPER, Brookfield, CT, USA) in their homecage over 5 weeks. Water and ethanol volume consumption were measured after each session (24 h/session) and total consumption was calculated (later referred to as noncued consumption). To induce ethanol dependence, 48 h after the last homecage drinking session, rats were assigned to receive either ethanol vapour treatment (*n* = 34) or control air treatment (*n* = 42), matched based on drinking levels during homecage induction. Vapour rats received 10 consecutive days (14 h on:10 h off) of chronic intermittent exposure to ethanol vapour and air rats received air only for the same duration. Rats remained in their homecage during vapour exposure. Blood (20 μL) was taken from the lateral saphenous vein under isoflurane anaesthesia every 3 days (3 times total) to measure blood ethanol content. 6–8 h after the final exposure session, physical withdrawal signs were measured to establish alcohol dependence. After 2 weeks of recovery, rats again received intermittent (MWF) access to 15E in their homecage over 2 weeks.

### 2.3 Alcohol Conditioning

All conditioning phases took place in chambers (30.5 × 24.1 × 29.2 cm, Med Associates Inc., Fairfax, VT, USA) enclosed in light‐ and sound‐attenuating boxes equipped with exhaust fans (Med Associates Inc., Fairfax, VT, USA) and digital video cameras to record all sessions for offline analysis. Each chamber was equipped with a house light, which served as the conditioned stimulus, and a bottle assembly with a retractable sipper dispensing 15E. Intertrial intervals were always variable 120–320 s. Rats underwent sipper training where they had access to the sipper with 15E for 30 min without the house light prior to receiving 12 consecutive daily conditioning sessions of 8 trials each. For each trial, a light cue was presented 10 s prior to a 10‐s 15E sipper presentation (20 s total) after which the light cue turned off and the sipper was retracted, co‐terminating the trial (Figure 1D). All conditioning sessions lasted approximately 40 min. Ethanol consumption was recorded each session, and total consumption calculated (later referred to as cued consumption).

### 2.4 Extinction and Retrieval‐Extinction

One day after the final conditioning session, rats were assigned to receive either standard extinction (*n* = 36) or retrieval‐extinction (*n* = 40). In both groups, rats received 14 daily consecutive sessions. In the extinction group, rats were first exposed to the conditioning chamber before returning to their homecage for 1 h. They then received 12 trials of light‐cue pairings with a retractable empty sipper. In the retrieval‐extinction group, rats received an isolated light‐cue trial with a retractable empty sipper in the conditioning chamber before returning to their homecage for 1 h. They then received 11 trials of light‐cue pairings with a retractable empty sipper. All retrieval/chamber exposure sessions lasted ~10 min, and all extinction sessions lasted approximately 60 min.

### 2.5 Long‐Term Memory Test

Two days after the last extinction session, rats were returned to the conditioning chambers for an LTM test. They received four trials of light‐cue pairings with a retractable empty sipper. Sessions lasted approximately 20 min.

### 2.6 Behavioural Scoring of Alcohol‐Seeking Behaviour

Sipper licks were detected and recorded during each trial presentation using Med Associates Inc. software (Fairfax, VT, USA). Sipper site approach was defined as approaching and/or exploring the sipper port and was sampled once every 1.25 s during each 20‐s trial. Approach data were manually scored by blind judges (≥ 95% interrater reliability) and are reported for the first 10 s of each trial (light only) and second 10 s of each trial (light + sipper) as a percentage of observations with approach behaviour.

### 2.7 CO_2_ Challenge

**FIGURE 2:**
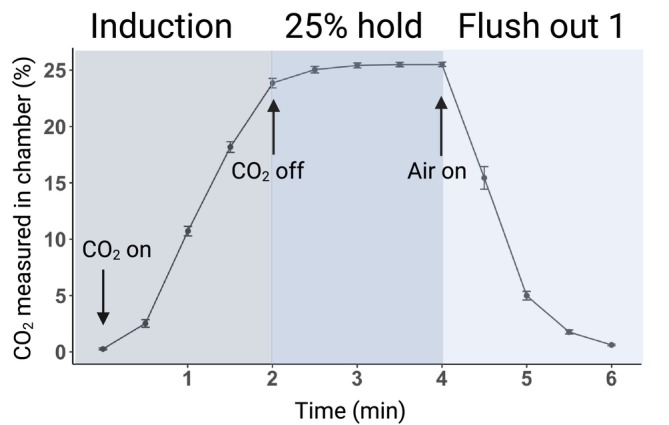
CO_2_ concentration measured in the chamber during induction, 25% hold, and flush out phases of the CO_2_ challenge. Mean (± SEM) as measured by a device located inside the chamber. Note that the concentration reaches a peak of approximately 25%.

Hereafter, CO_2_ challenge will refer to the assay and CO_2_ reactivity will refer to the behavioural output. As described in our previous publications, the assay took place in a plexiglass chamber (30.5 × 30.5 × 61 cm) that allows the control of gas flow as shown in Figure 2. Thirty seconds after the rat was placed in the chamber (baseline), an infusion of normoxic air blended with hypercarbic gas (25% CO_2_) was released into the chamber for 2 min (induction phase). Then, gas flow was held constant at 25% CO_2_ for 2 min (hold phase), followed by 4 min of infusion with normoxic air (flush‐out phases 1 and 2). Each rat remained in the chamber for an additional 2 min before returning to its home cage. All sessions were recorded and analysed offline for the following four behaviours: ambulation (time spent moving around; any displacement of the paws), grooming (time spent grooming), rearing (number of times rat stands on rear legs) and laboured breathing (deep and long breaths noticeable from movements of the torso). The four CO_2_ reactivity behaviours across the four phases yielded 16 variables or subcomponents. All videos were manually scored using Behavioural Observation Research Interactive Software (BORIS; [36]).

### 2.8 Euthanasia and Brain Preparation

One hour following the CO_2_ challenge, rats received a lethal peritoneal injection of a pentobarbital and phenytoin solution (Euthasol; Virbac Animal Health, 1 mL/200 g). Female rats were vaginally lavaged with 40 μL saline to determine estrous cycle stage using cytological examination [37]. The chest cavity was opened, and in female rats, uterine horn width was measured. All rats were intracardially perfused with phosphate buffered saline (PBS), followed by 4% paraformaldehyde (PFA). Brains were extracted and stored in 4% PFA for 24–48 h before transfer to a solution of 30% phosphate‐buffered sucrose for 48 h. Brains were then flash frozen in powdered dry ice and stored at −80°C until sectioning. For each rat, the entire brain was sectioned coronally on a sliding microtome at a thickness of 40 μm and stored in four series. One series was stored in PBS at 10°C for up to 1 week until immunohistochemical processing could occur, and the other three series were stored in cryoprotectant at −20°C.

### 2.9 Immunohistochemistry

For each rat, sections from one series containing the LH were dual stained for c‐Fos and orexin‐A protein expression in batches of six to eight brains. All rinses were in PBS and all incubations were in solutions of PBS with 0.2% detergent (Triton‐X; Fisher Scientific), unless otherwise noted. Free floating sections were rinsed after removal from storage and incubated overnight in mouse orexin A antibody (1:1000 dilution; R&D Systems) at 5°C. Sections were then rinsed and incubated in secondary antibody (1:1000 dilution; Donkey Anti‐Mouse 568 (red); Alexa Fluor, Abcam) for 1 h before being rinsed and incubated in 1% detergent in PBS for 1 h. Sections were washed and blocked in 2% normal goat serum (Vector Laboratories) for 1 h before incubation in rabbit c‐Fos antibody (1:1000 dilution; Immunostar) and 2% normal goat serum for 3 days at 5°C. Finally, sections were rinsed and incubated in secondary antibody (1:1000 dilution; Goat Anti‐Rabbit 488 (green); Alexa Fluor, Abcam) overnight at 5°C. Sections were then rinsed, mounted and cover slipped with a fluorescence mounting medium (Prolong Gold Antifade Mountant; Invitrogen). Slides were allowed to dry at room temperature for 24–48 h and then stored at 5°C until imaging.

### 2.10 Imaging and Quantification

**FIGURE 3:**
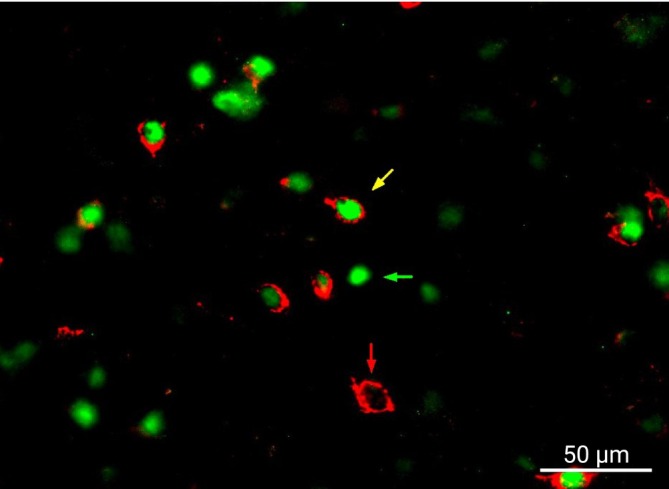
c‐Fos and orexin in the lateral hypothalamus. Representative image of c‐Fos (green) and orexin (red) immunohistochemistry. Red arrow indicates cell labelled with orexin only, green arrow indicates cell labelled with c‐Fos only, and yellow indicates cell co‐labelled with c‐Fos and orexin.

Sections were visualized on a fluorescence microscope (Axio Scope A1; Zeiss) under a 10× objective through an eyepiece with a magnification of 12.5× to identify the LH in accordance with Paxinos and Watson [38]. Bilateral images were taken under a 20× objective of sections between −2.3 and −3.3 from Bregma under fluorescent wavelengths for the secondary antibodies. Images were merged and cells that expressed c‐Fos, orexin or both were counted with blinding to grouping variables using ImageJ (NIH). Counts were averaged across sections for each subject. Viable, bilateral images from three to five sections were required for a rat to be included in the analysis. A representative image is shown in Figure 3. To account for potential batch effects, cell counts are reported as the ratio of co‐labelled c‐Fos + orexin cells to orexin cells. Raw counts of each cell type can be found in Table S1.

### 2.11 Statistical Analysis

All statistical analyses were performed using R and the following packages: rstatix, ggpubr and beset [39]. We conducted 2 × 2 × 2 ANOVAs on each subcomponent of CO_2_ reactivity with sex, vapour group and extinction group as between‐subjects factors. We also conducted a 2 × 2 × 2 ANOVA on orexin/c‐Fos colocalization with these factors. Post hoc Welch's *t* tests (to control for unequal sample sizes) were conducted as warranted. We do not report ANOVAs on LTM nor comprehensive analysis of the induction/dependence, conditioning and extinction phases of the experiment as those will be reported in a separate forthcoming manuscript.

We used the best subset approach to linear regression, which allows us to determine which of the 16 subcomponents of CO_2_ reactivity predicts the most variance in LTM. Linear regression allows us to take advantage of the whole range of values and minimize the amount of information lost (which occurs when variables are categorized into subgroups). The best subset approach chooses the linear model with the fewest predictors, highest predictive power and highest degree of cross‐sample replicability. This approach weighs the contribution of each variable's share of explained variance to ensure it is worth including in the model. We also used resampling (k‐fold cross‐validation where *k* = 10) on each possible combination of predictors to estimate how well it would predict new samples. To minimize selection bias, we also ran a nested cross‐validation, such that test error could be evaluated on a hold‐out sample that was not used to fit or select the best models [40, 41]. This provides a fairer estimate of the model's generalizability and allows us to analyse how sensitive the selection outcome was to randomized assignments of observations to cross‐validation folds. In other words, the selected model is the one with the fewest predictors that is best at predicting new data. We ran the best subset analysis separately for the extinction and retrieval‐extinction groups. We used all 16 subcomponents of CO_2_ reactivity, as well as sex and vapour group, as possible predictor variables of LTM. The first two trials of LTM were averaged together for sipper licks, approach during the light and approach during the light + sipper. Each of these was used as an outcome variable, and separate models were generated for the extinction and retrieval‐extinction groups.

We evaluated the following relationships using Pearson's correlations: CO_2_ reactivity and LTM, CO_2_ reactivity and orexin/c‐Fos colocalization, orexin/c‐Fos colocalization and LTM. We also evaluated the relationships between the three measures of LTM. Lastly, we examined the relationship between cued and noncued alcohol consumption (reported as g/kg) and orexin/c‐Fos colocalization and performed 2 × 2 × 2 ANOVAs for each with sex, vapour group and extinction group as between‐subjects factors.

## 3 Results

### 3.1 Main Analyses

#### 3.1.1 CO_2_ Reactivity

**FIGURE 4:**
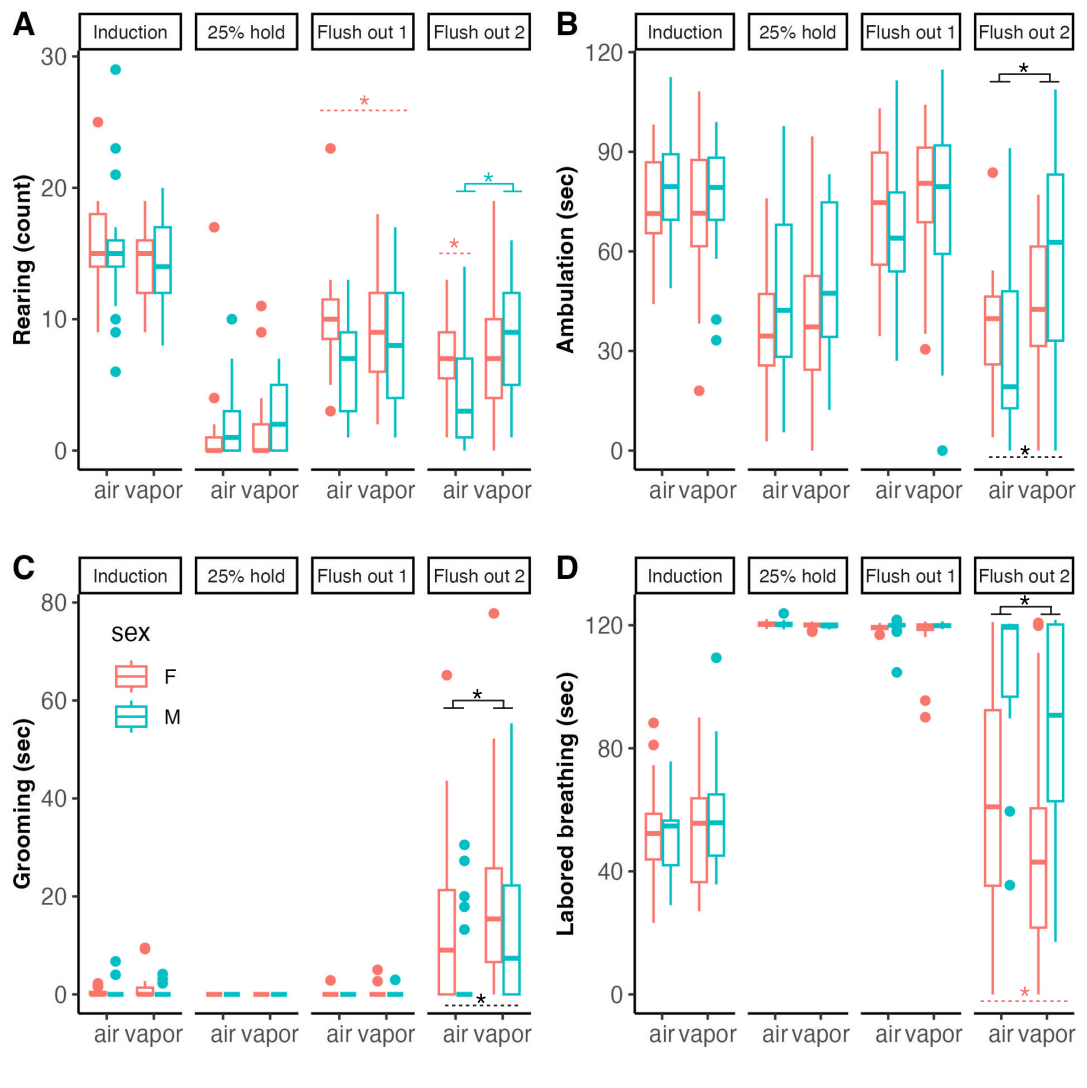
Distribution of CO_2_ reactivity behaviours. Boxplots of the behaviours that were analysed (rearing, ambulation, grooming, laboured breathing) during each phase (induction, 25% hold, flush out 1, flush out 2) during the CO_2_ challenge. This yielded 16 behavioural subcomponents that were entered as possible predictors (along with sex and vapour group) of alcohol‐seeking behaviour after extinction or retrieval‐extinction in our model. Vapour rats displayed significantly more ambulation and grooming and less laboured breathing during flush out 2 than air rats. Female rats displayed significantly more rearing during flush out 1 and male rats displayed significantly more laboured breathing during flush out 2. For flush out 2, in the air group females displayed significantly more rearing than males and among the males, the vapour group displayed significantly more rearing than the air group, and the retrieval‐extinction group displayed significantly more grooming but less ambulation than the extinction group. Solid black lines indicate differences between air groups, solid‐coloured lines indicate differences between air groups among males, coloured dashed lines indicate sex differences and black dashed lines indicate differences between extinction groups. *p < 0.05.

We quantified the following behaviours during a CO_2_ challenge in rats: ambulation, rearing, laboured breathing and grooming. This yielded 16 behavioural subcomponents of CO_2_ reactivity. The distribution of these behavioural subcomponents during the CO_2_ challenge was visualized between sex and vapour groups using boxplots (Figure 4). Two female rats (both from the air group) had seizures during the flush out phase of the CO_2_ challenge and were excluded from analysis. A 2 × 2 × 2 ANOVA (sex × vapour group × extinction group) was run for each subcomponent of CO_2_ reactivity. Significant interactions and main effects are summarized in Table S2.

Post hoc *t* tests revealed that vapour rats displayed significantly more ambulation (*t*(62.10) = 2.18, *p* = 0.033) and grooming (*t*(59.65) = 2.10, *p* = 0.040) and less laboured breathing (*t*(66.24) = 2.55, *p* = 0.013) during flush out 2 than air rats. Female rats displayed significantly more rearing during flush out 1 (*t*(67.69) = 2.61, *p* = 0.011) and male rats displayed significantly more laboured breathing during flush out 2 (*t*(71.84) = 5.54, *p* < 0.001). For flush out 2, in the air group, females displayed significantly more rearing than males (*t*(36.59) = 2.89, *p* = 0.006), and among the males, the vapour group displayed significantly more rearing than the air group (*t*(22.19) = 2.80, *p* = 0.01), and the retrieval‐extinction group displayed significantly more grooming (*t*(57.93) = 2.40, *p* = 0.020) but less ambulation than the extinction group (*t*(71.25) = 2.13, *p* = 0.037). Significant post hoc *t* tests within either extinction group are discussed and visualized in Supporting Information Figure 1. No other post hoc *t* tests were significant.

#### 3.1.2 CO_2_ Reactivity Differentially Predicts Alcohol‐Seeking Behaviour After Extinction and Retrieval‐Extinction

To determine whether CO_2_ reactivity predicts the return of alcohol‐seeking behaviour after extinction or retrieval‐extinction, we used behavioural CO_2_ reactivity (along with sex and vapour group) as predictors of long‐term alcohol memory using the best subset approach to linear regression to estimate the best model within 1 standard error, with the lowest cross‐validation error and with the fewest number of predictors. We report two *R* ^2^ statistics for each model: the train‐sample *R* ^2^ (the unadjusted *R* ^2^ obtained by fitting the model to the full dataset) and the cross‐validated *R* ^2^ (the *R* ^2^ obtained by predicting random holdout examples and used to select the best model). This analysis was run separately for extinction and retrieval‐extinction to determine whether there is a differential predictive effect of CO_2_ reactivity in the two groups and to remain consistent with our prior work. For each group, we computed a model for each measurement of LTM: sipper licks, approach during the light and approach during the light + sipper.

**FIGURE 5:**
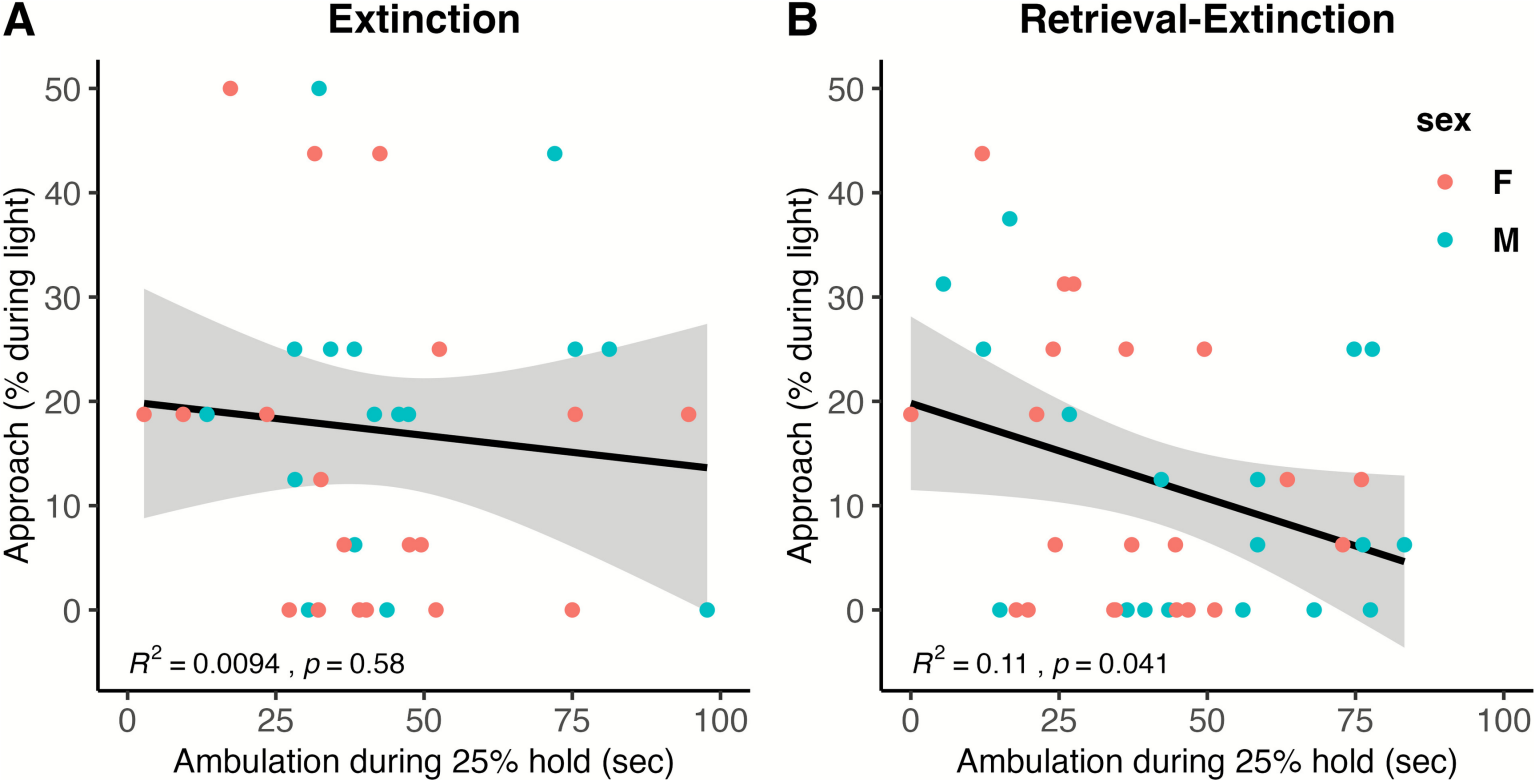
Relationship of the strongest predictor of LTM as measured by approach behaviour during the light cue. In the extinction group (A), CO_2_ reactivity does not reliably predict LTM measured this way. In the retrieval‐extinction group (B), ambulation during 25% hold predicts 13% of the variability in the current sample and is expected to predict 6% of the variability in future samples.

When LTM was measured using approach during the light as the outcome variable, in the extinction group, the null model was selected 81% of the time, and no other models were selected more than 3% of the time. In other words, CO_2_ reactivity did not predict LTM in the extinction group when measured this way. In the retrieval‐extinction group, the null model was selected 33% of the time, followed by a model with one predictor, ambulation during 25% hold, selected 26% of the time. The train sample *R* ^2^ was 0.129 and the CV‐tune *R* ^2^ was 0.06, meaning that this model predicts 12.9% of the variability in the current sample and is expected to predict 6% of the variability in future samples. The relationship of ambulation during 25% hold with LTM approach during the light is shown in Figure 5A for the extinction group and Figure 5B for the retrieval‐extinction group.

**FIGURE 6:**
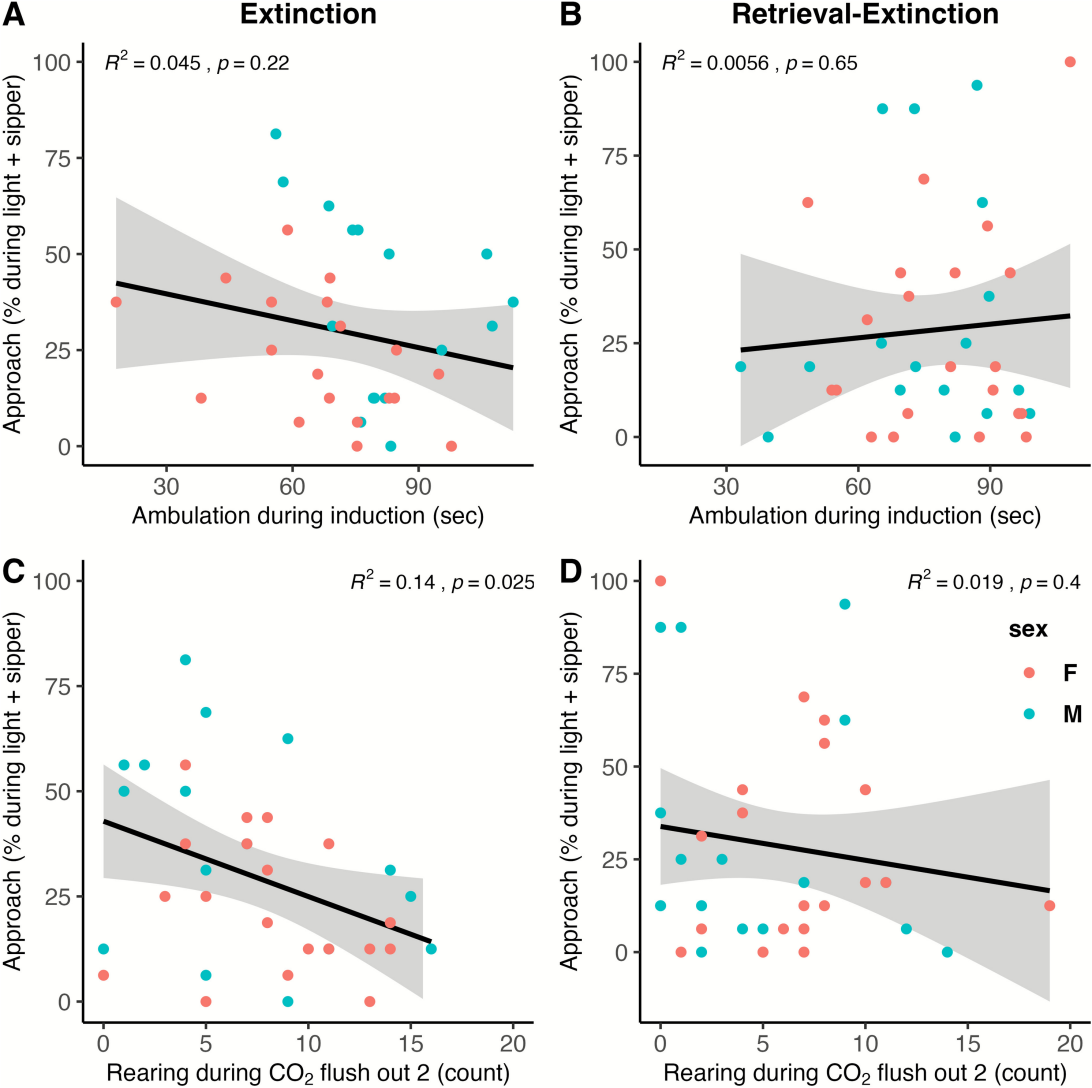
Relationship of the strongest predictors of LTM as measured by approach behaviour during the light + sipper cue. In the extinction group, ambulation during induction (A), rearing during flush out 2 (C) and sex predict 17% of the variability in the current sample and are expected to predict 10% of the variability in future samples. In the retrieval‐extinction group, CO_2_ reactivity does not predict LTM measured this way. The relationships with ambulation during induction (B) and rearing during flush out 2 (D) are shown.

When LTM was measured using approach during the light + sipper as the outcome variable, in the extinction group, a model with rearing during flush out 2 was selected 30% of the time, followed by a model with sex and ambulation during induction selected 21% of the time. The train sample *R* ^2^ was 0.167 and the CV‐tune *R* ^2^ was 0.097, meaning that 16.7% of the variability was predicted in the current sample and is expected to predict 9.7% of the variability in future samples. In the retrieval‐extinction group, the null model was selected 88% of the time, and no other models were selected more than 7% of the time. In other words, CO_2_ reactivity did not predict LTM in the retrieval‐extinction group when measured this way. The relationships of ambulation during induction and rearing during flush out 2 with LTM approach during the light + sipper are shown in Figure 6A and C for the extinction group and Figure 6B and D for the retrieval‐extinction group.

**FIGURE 7:**
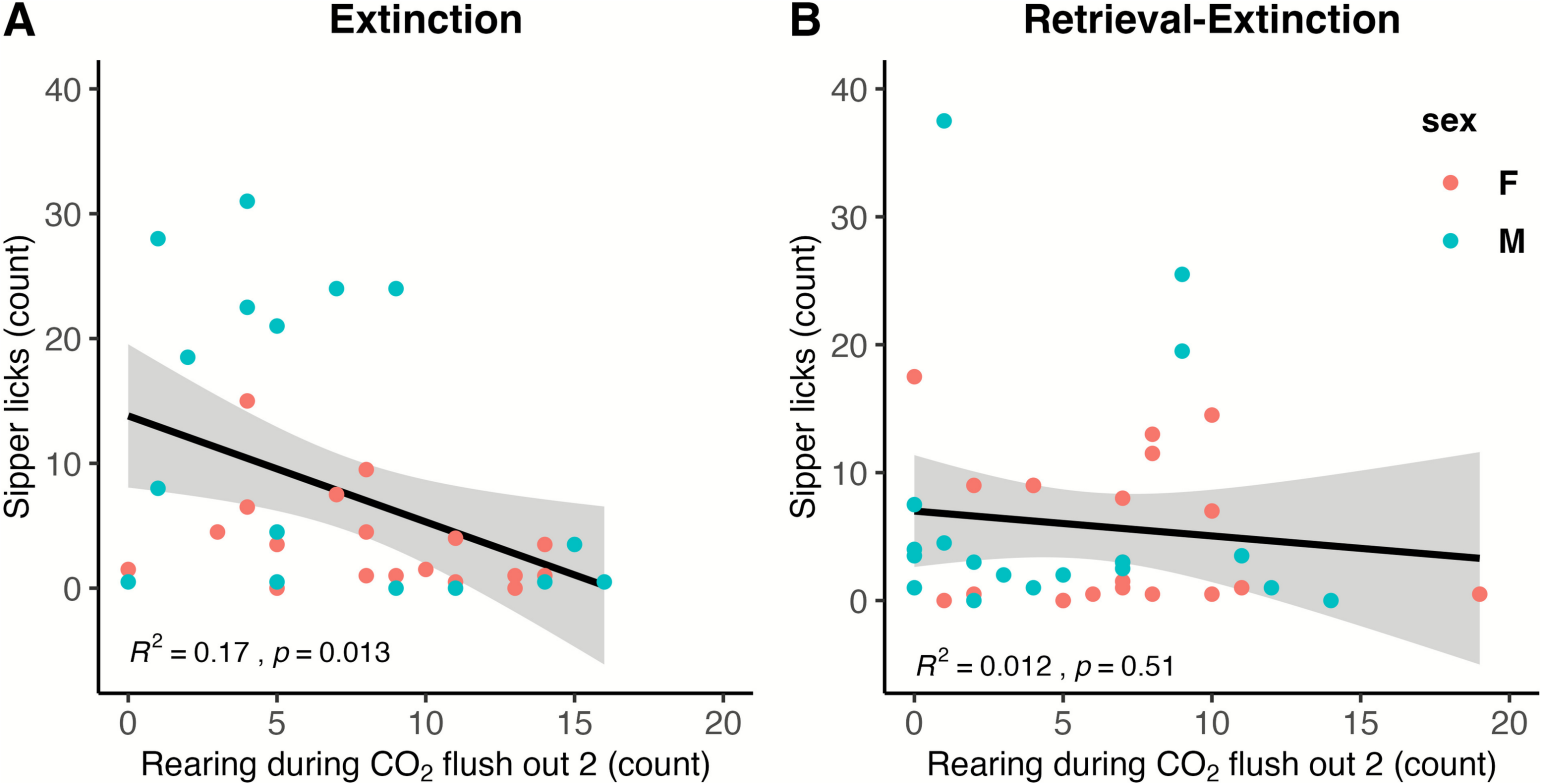
Relationship of the strongest predictor of LTM as measured by sipper licks. We used the best subset approach to linear regression to determine which components of CO_2_ reactivity would predict the return of alcohol‐seeking behaviour. In the extinction group (A), sex and rearing during flush out 2 explain 17% of the variability in the current sample and are expected to predict 7.5% of the variability in future samples. In the retrieval‐extinction group (B), no reliable predictors were identified.

When LTM was measured using sipper licks as the outcome variable, in the extinction group, a model with sex was selected 35% of the time, followed by a model with rearing during flush out 2 selected 19% of the time, and a model with both predictors was selected 15% of the time. The train sample *R* ^2^ was 0.166 and the CV‐tune *R* ^2^ was 0.0749, meaning that the model predicts 16% of the variability in the current sample and is expected to predict 7.5% of the variability in future samples. In the retrieval‐extinction group, no reliable predictors were identified. The relationship of rearing during flush out 2 with LTM sipper licks is shown in Figure 7A for the extinction group and Figure 7B for the retrieval‐extinction group.

#### 3.1.3 No relationship Between Orexin/c‐Fos Colocalization and CO_2_ Reactivity or LTM

We hypothesized that subcomponents of CO_2_ reactivity that were predictive of LTM would also be predictive of orexin/c‐Fos colocalization. We examined whether there were significant relationships between orexin/c‐Fos colocalization and rearing during flush out 2, ambulation during 25% hold, or ambulation during induction, and there were not (all *p*s > 0.15). These relationships are shown in Supporting Information Figure 2. We hypothesized that orexin/c‐Fos colocalization would be predictive of LTM. We examined whether there were significant relationships between orexin/c‐Fos colocalization and the three measures of LTM, and there were not (all *p*s > 0.18). These relationships are shown in Supporting Information Figure 3.

#### 3.1.4 In the Extinction Group, Male Rats Have Significantly Higher Orexin/c‐Fos Colocalization Than Female Rats

**FIGURE 8:**
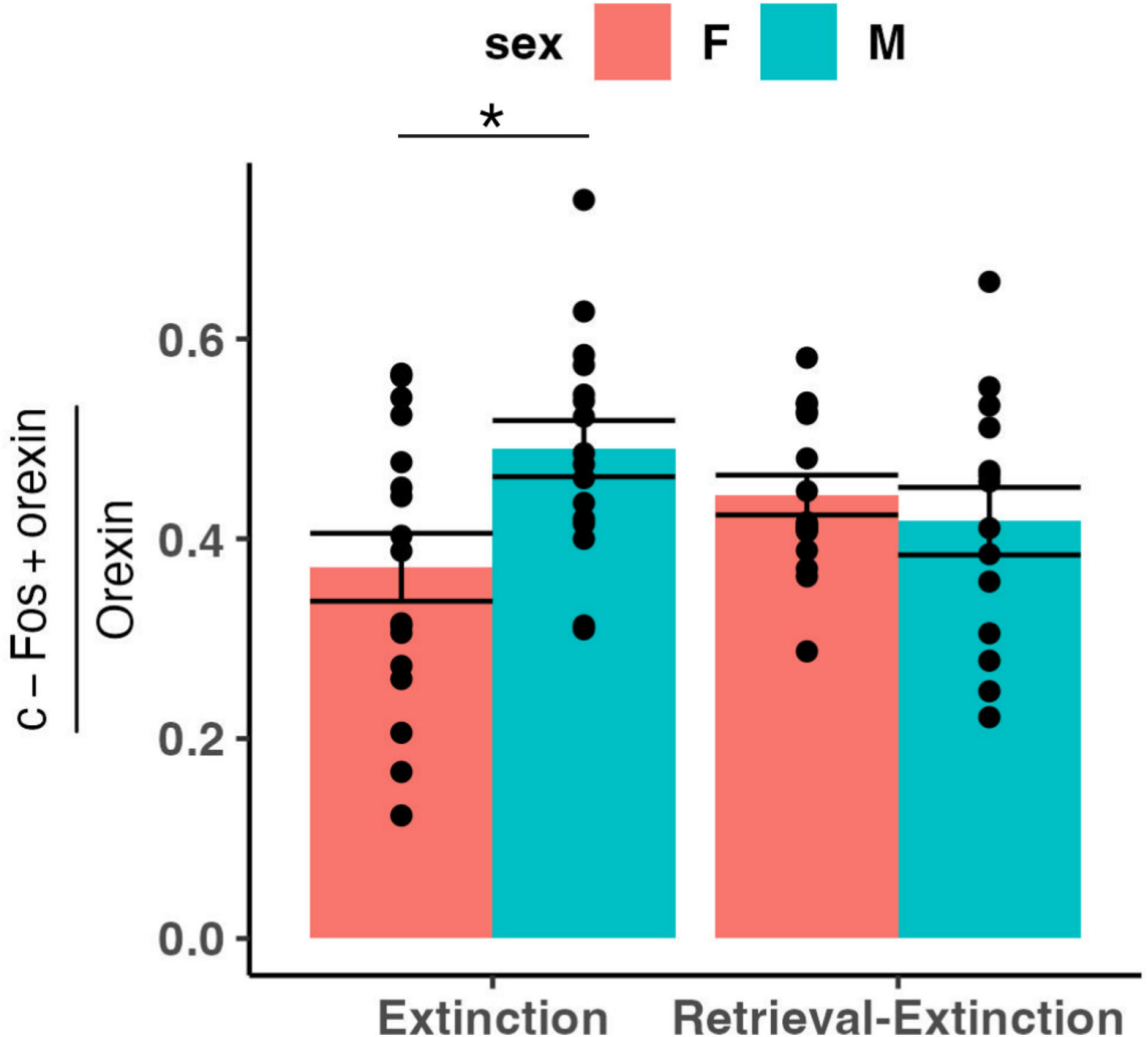
CO_2_‐induced orexin/c‐Fos colocalization. The mean ratio of co‐labelled c‐Fos and orexin cells to orexin cells is shown, ± SEM. In the extinction group, male rats had significantly higher orexin/c‐Fos colocalization than female rats. *p < 0.05.

To determine whether there was an effect of sex, vapour group or extinction group on orexin/c‐Fos colocalization, we conducted a 2 × 2 × 2 ANOVA. There was a significant interaction of sex and extinction group (*F*(1,55) = 6.50, *p* = 0.014). Post hoc tests showed a significant main effect of sex in the extinction group (*F*(1,31) = 7.15, *p* = 0.012), but not the retrieval‐extinction group (*F*(1,28) = 0.470, *p* = 0.50). In the extinction group, males had significantly more orexin/c‐Fos colocalization than females (*t*(30.27) = 2.69, *p* = 0.011). Orexin/c‐Fos colocalization in these groups is shown in Figure 8. To determine whether this difference might be due to differences in the number of orexin‐expressing or c‐Fos‐expressing cells, we conducted 2 × 2 × 2 ANOVAs on orexin and c‐Fos cell counts. For orexin, there were significant main effects of sex (*F*(1,55) = 5.99, *p* = 0.018) and extinction group (*F*(1,55) = 5.01, *p* = 0.029) but follow‐up *t* tests were not significant. For c‐Fos, there were no significant main effects nor interactions. The difference in orexin/c‐Fos colocalization was not due to the estrous cycle in females, as a 2 × 2 × 4 ANOVA (with vapour group, extinction group and estrous cycle stage as factors) was not significant, nor was a 2 × 2 × 2 ANOVA when stages were categorized as “high” (protestrus and estrus) or ‘low’ (metestrus and diestrus) hormonal states. A regression model with vapour group, extinction group and uterine horn width as independent variables was not significant.

### 3.2 Exploratory Analyses

#### 3.2.1 Approach During the Light Predicts Approach During the Light + Sipper in the Retrieval‐Extinction Group but Not the Extinction Group During LTM

**FIGURE 9:**
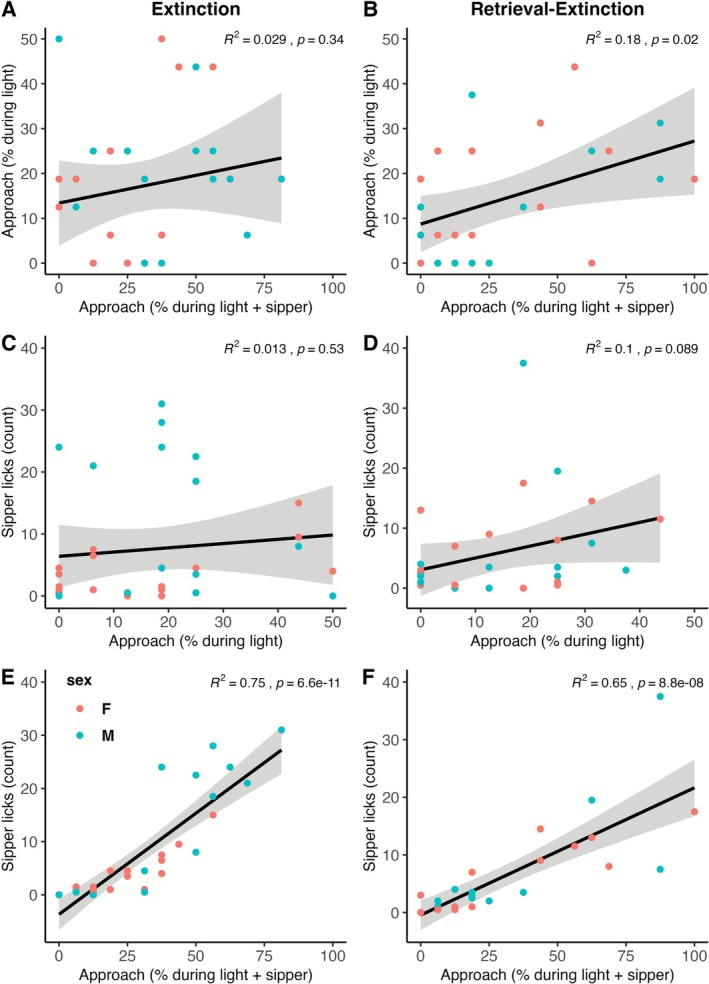
Relationships between the three measures of LTM. The relationship between approach during the light and approach during the light and sipper is not significant in the extinction group (A) but is significant in the retrieval‐extinction group (B). The relationship between sipper licks and approach during the light is not significant in the extinction group (C) nor the retrieval‐extinction group (D). The relationship between sipper licks and approach during the light and sipper is significant in both the extinction group (E) and the retrieval‐extinction group (F).

Exploratory analyses were conducted to determine why CO_2_ reactivity predicted LTM as measured by sipper licks and approach during the light + sipper in the extinction group but not the retrieval‐extinction group, and predicted LTM as measured by approach during the light in the retrieval‐extinction group but not the extinction group. As expected, in both the extinction and retrieval‐extinction groups, there was a significant relationship between sipper licks and approach during the light + sipper (*R* ^2^s > 0.65, *p*s < 0.0001). In the extinction group, the relationships between sipper licks and approach during the light, and between approach during the light and approach during the light + sipper, were not significant (*p*s > 0.34). In the retrieval‐extinction group, the relationship between sipper licks and approach during the light was not significant (*p* = 0.089), but the relationship between approach during the light and approach during the light + sipper was significant (*R* ^2^ = 0.18, *p* = 0.02). These relationships are shown in Figure 9.

#### 3.2.2 Relationship Between Cued Ethanol Consumption and Orexin/c‐Fos Colocalization in the Extinction Group

**FIGURE 10:**
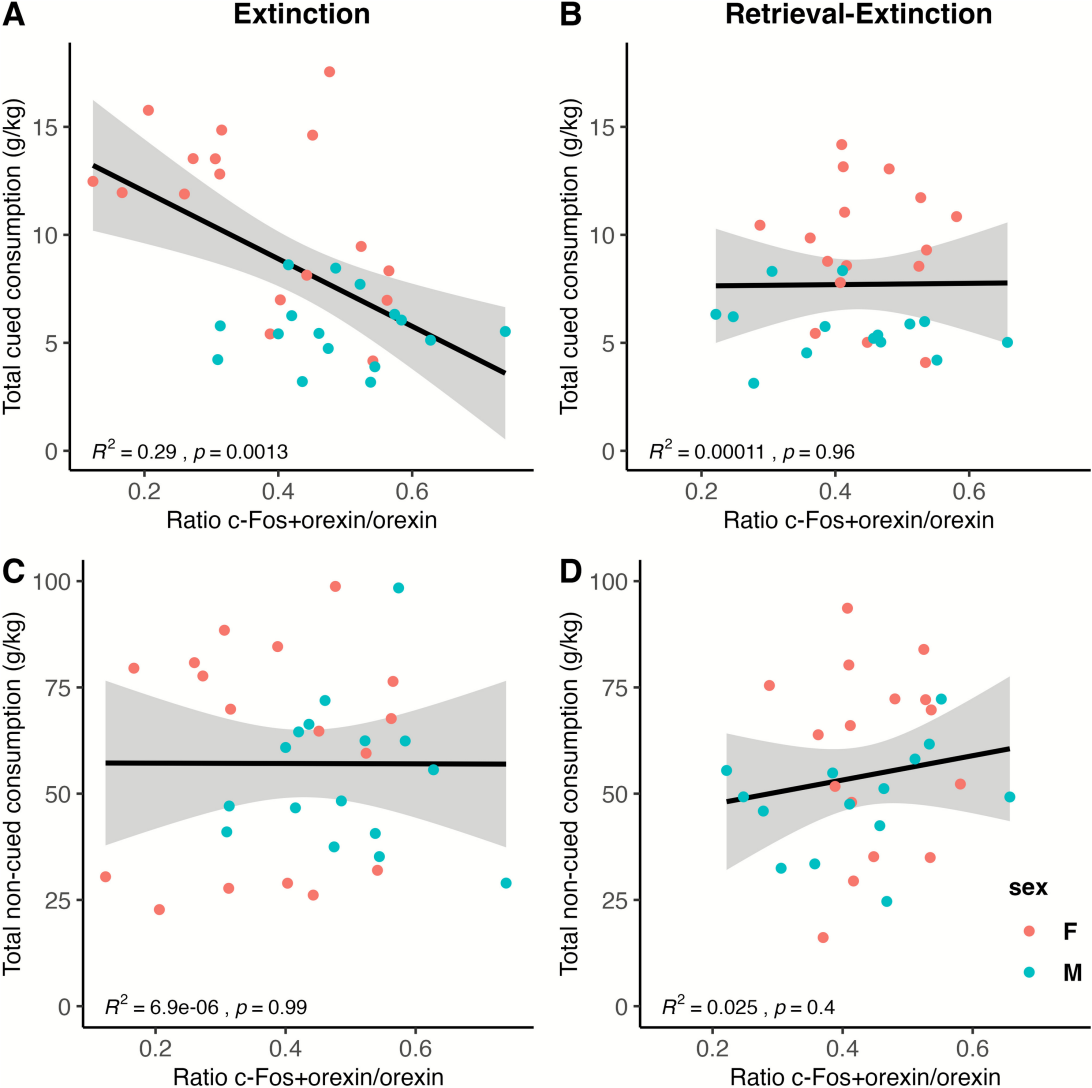
Relationships between CO_2_‐induced orexin/c‐Fos colocalization and cued and noncued ethanol consumption. There was a significant negative relationship between total cued ethanol consumption during the conditioning phase and orexin/c‐Fos colocalization in the extinction group (A) but not the retrieval‐extinction group (B). There was no significant relationship between total noncued ethanol consumption during the 5‐week homecage drinking phase in the extinction group (C) nor the retrieval‐extinction group (D).

Exploratory analyses were conducted to determine whether there was a relationship between voluntary ethanol consumption and CO_2_‐induced orexin/c‐Fos colocalization. There was a significant negative association between total cued ethanol consumption during the conditioning phase and orexin/c‐Fos colocalization in the extinction group (*R* ^2^ = 0.29, *p* = 0.0013) but not the retrieval‐extinction group (*R* ^2^ < 0.001, *p* = 0.96). There was no relationship between total noncued ethanol consumption during the 5‐week homecage drinking phase and orexin/c‐Fos colocalization in either group (*R* ^2^s < 0.025, *p*s > 0.4). These relationships are shown in Figure 10. There were no relationships between cued or noncued consumption and orexin cell count or c‐Fos cell count in either group. A 2 × 2 × 2 ANOVA revealed a significant main effect of sex for cued consumption, with females drinking almost twice as much as males (*t*(44.45) = 6.99, *p* < 0.0001).

## 4 Discussion

Here, we sought to determine whether the predictive power of CO_2_ reactivity might extend to alcohol‐seeking behaviour after extinction or retrieval‐extinction in alcohol dependent and nondependent male and female rats. We also examined the relationship between CO_2_ reactivity, return of alcohol‐seeking behaviour and orexin/c‐Fos colocalization. We found that CO_2_ reactivity differentially predicted LTM after extinction and retrieval‐extinction. We did not find any relationships between orexin/c‐Fos colocalization and CO_2_ reactivity or LTM. There was no effect of alcohol dependence on any of these relationships.

We extended our previous findings that CO_2_ reactivity predicts extinction memory for fear and food cues and that CO_2_ reactivity predicts fear expression after retrieval‐extinction, albeit to a lesser degree. Here, the strongest predictor of LTM of alcohol extinction was rearing during flush out 2, which was negatively associated with LTM. In our prior study of food extinction memory, we also found a significant negative association between rearing during flush out 2 and LTM [21]. This suggests that we may have identified a common predictor of extinction memory for reward cues. In our prior studies of fear extinction and retrieval‐extinction memory, we found a significant negative association between rearing during flush out 1 and LTM for extinction [20, 34]. We also found that variability in LTM was explained by different predictors for the extinction and retrieval‐extinction groups and that less variability was explained by CO_2_ reactivity in the retrieval‐extinction group [34]. That CO_2_ reactivity differentially predicts LTM after extinction and retrieval‐extinction may reflect their distinct yet overlapping underlying neural mechanisms [42, 43]. One important difference between our current and previous work is that here, we only conducted a CO_2_ challenge after extinction, whereas we previously also conducted the CO_2_ challenge before extinction. However, in both designs, behavioural reactivity during the first CO_2_ challenge was associated with extinction memory, and a recent analysis suggests that a prior CO_2_ challenge affects subsequent CO_2_ reactivity. Nevertheless, it will be important to determine whether CO_2_ reactivity predicts subsequent extinction memory in future studies.

### 4.1 Extinction and Retrieval‐Extinction Result in Differential Prediction by CO_2_ Reactivity and Relationships Among Elements in the CS‐US Associative Chain

In the present study, LTM was quantified using three measures, each of which was differentially predicted by CO_2_ reactivity across the two treatment groups. In the extinction group, CO_2_ reactivity predicted LTM when quantified by sipper licks or approach during the light + sipper. In the retrieval‐extinction group, CO_2_ reactivity predicted LTM when quantified by approach during the light. It is not surprising that CO_2_ reactivity is similarly predictive for sipper licks and approach during the light + sipper in the extinction group. Indeed, these behaviours are not mutually exclusive when the sipper is present and were highly correlated in both groups. However, it is interesting that the relationship between approach during the light and approach during the light + sipper was significant in the retrieval‐extinction group but not the extinction group and that the relationship between approach during the light and sipper licks was not significant in either group. This contrasts with prior work from our lab showing that in well‐trained rats, greater approach to the light predicts greater sipper licks during conditioning [44, 45]. This suggests that extinction and retrieval‐extinction result in a differential dissociation between cue‐driven and consummatory behaviour. In the extinction group, the presentation of the *sipper* initiates approach, whereas in the retrieval‐extinction group, the presentation of the *light* initiates approach. Across both conditioning and extinction, the light always precedes the sipper, but the sipper only precedes alcohol availability during conditioning. When retrieval‐extinction is successful, the original CS‐US memory is updated with a CS‐noUS memory—or in this case, the light‐sipper‐alcohol memory is updated with a light‐sipper memory, and it is approach to the light CS that is predicted by CO_2_ reactivity in this group. When retrieval‐extinction is not successful, then, we might expect to see a pattern of behaviour consistent with that of a light‐sipper‐alcohol memory. In contrast, with extinction, the light‐sipper‐alcohol memory competes for expression with the light‐sipper‐noAlcohol memory. Because the light always predicts the sipper, it is the sipper‐alcohol and sipper‐noAlcohol memories that compete for expression, which would in this case be measured by sipper licks—precisely what is predicted by CO_2_ reactivity in this group. Thus, it appears that retrieval‐extinction training results in increased relevance of the light CS (the most distal element in the associative chain to the US), whereas extinction training results in increased relevance of the sipper (the most proximal element in the associative chain to the US), leading them to drive behaviour that is ultimately predicted by CO_2_ reactivity.

Like approach to the light, conditioned orienting is also a form of cue‐driven behaviour. Previous work from our lab has found that retrieval‐extinction is effective at preventing the return of conditioned food‐seeking behaviour only in rats that display high conditioned orienting to the light CS and that neither retrieval‐extinction nor extinction attenuates conditioned orienting [29]. Both conditioned orienting and retrieval‐extinction are mediated by the central nucleus of the amygdala (CeA; [29, 46]). Appetitive extinction training activates neurons in the CeA that are critical for extinction expression [47]. Furthermore, mice with high behavioural sensitivity to CO_2_ show increased activation of the CeA (as measured by ΔFosB; [48]). We had hypothesized that CO_2_ reactivity and LTM would be predicted by orexin/c‐Fos colocalization, but the present results do not support that hypothesis. Instead, it may be that the predictive effect of CO_2_ reactivity reflects the degree of CeA engagement. Future work should examine the role of the CeA in alcohol extinction and retrieval‐extinction, as well as CO_2_ reactivity in rats.

### 4.2 Effects of Sex and Extinction Group on Orexin/c‐Fos Colocalization and Cue‐induced Alcohol Consumption, but No Relationship Between Orexin/c‐Fos Colocalization and CO_2_ Reactivity or Extinction Memory

We also assessed CO_2_ reactivity and its relationship to extinction memory and orexin/c‐Fos colocalization in female rats for the first time. There was an overall sex difference in rearing during flush out 1 and a sex difference in rearing during flush out 2 in the air group. In humans, there are also sex differences in CO_2_ reactivity, with women reporting higher levels of fear and panic following CO_2_ challenge than men [49]. Rearing during flush out 1 was identified as a significant predictor of fear extinction memory in two samples [20, 34] and rearing during flush out 2 was identified as a significant predictor of reward extinction memory both here and in a food conditioned sample [21]. These findings suggest that there are sex differences in subcomponents of CO_2_ reactivity that predict extinction memory. However, these differences do not appear to preclude the utility of CO_2_ reactivity to predict extinction memory in female rats. While sex was a significant predictor of extinction memory, this only indicates that male rats are more likely to have worse extinction memory, which is consistent with existing literature [50].

Orexin/c‐Fos colocalization was not significantly related to CO_2_ reactivity or extinction memory, but there was a significant interaction between sex and extinction group for orexin/c‐Fos colocalization, with female rats showing less orexin/c‐Fos colocalization than male rats in the extinction group. This conflicts with prior reports that female rats have significantly more food‐ and stress‐induced orexin/c‐Fos colocalization than male rats [51, 52] but may be related to the rats' pattern of alcohol consumption. Female rats drank nearly twice as much as male rats (as a function of body weight) over the course of alcohol conditioning, and there was a significant negative correlation between cued alcohol consumption and orexin/c‐Fos colocalization. We have previously found a negative relationship between orexin/c‐Fos colocalization and food consumption (though only in male, not female rats) [45]. Prior studies have found that voluntary binge‐like ethanol consumption reduces the number of orexin neurons and orexin mRNA expression in male mice [53, 54]. Male and female rats with a history of alcohol dependence via 6 weeks of chronic intermittent exposure who received alcohol self‐administration training and extinction during weeks 4–6 show increased orexin mRNA expression compared to their nondependent counterparts [55]. Orexin mRNA expression in the LH is decreased by chronic voluntary ethanol consumption in a dose‐dependent manner, but involuntary binge ethanol consumption increases orexin expression at a low dose, but not a high dose [56]. While there was no effect of vapour group on orexin/c‐Fos colocalization or number of orexin cells, it may be that orexin/c‐Fos colocalization was downregulated by repeated sessions of alcohol conditioning and extinction. Orexin antagonism decreases operant alcohol self‐administration [16, 57, 58, 59] and prevents spontaneous recovery, renewal and reinstatement of ethanol seeking and consumption [14, 15, 16, 17, 18]. Increased orexin activity is also associated with greater alcohol seeking during renewal and reinstatement [13, 14]. It is unknown if the orexin system plays a role in retrieval‐extinction but given its role in alcohol conditioning and extinction memory, and the female rats' increased cued consumption, it seems plausible that repeated engagement of the system could have resulted in its downregulation among the female rats in the extinction group, as well as the significant negative relationship between cued alcohol consumption and orexin/c‐Fos colocalization in the extinction group.

## 5 Conclusions

We found that the predictive power of CO_2_ reactivity extends to alcohol‐seeking behaviour after both extinction and retrieval‐extinction in alcohol dependent and nondependent male and female rats, while its relationship with orexin/c‐Fos colocalization does not. Along with our previous work, the current study bolsters the idea that CO_2_ reactivity could be used as a screening tool to identify patients who are not likely to benefit from CET and that a mechanistically distinct treatment, potentially including a retrieval‐extinction–based approach, might be a useful alternative for these patients [21, 34]. Ideally, such a screening tool could be used to assign patients to one treatment or another. Here, more ambulation during induction and rearing during flush out 2 was associated with less return of alcohol‐seeking behaviour after extinction, and more ambulation during 25% hold was associated with less return of alcohol‐seeking behaviour after retrieval‐extinction. While CO_2_ challenge is safe, easy and inexpensive to administer in humans, it is unclear whether or how these specific CO_2_ reactivity measures in rodents correspond to CO_2_ reactivity in humans. Future studies should examine whether a priori CO_2_ screening for treatment assignment can be used to prevent relapse in a greater number of individuals.

## Author Contributions

Study concept and design: Marie‐H. Monfils, Jason Shumake, Hongjoo Lee. Analysis and interpretation of data: Marissa Raskin, Marie‐H. Monfils, Jason Shumake. Drafting of the manuscript: Marissa Raskin and Marie‐H. Monfils. Critical revision of the manuscript for important intellectual content: Marissa Raskin, Marcelle Olvera, Kylee Smith, Roberto Cofresi, Jason Shumake, Michael Telch, Michael Otto, Jasper Smits, Rueben Gonzales, Hongjoo Lee, Marie Monfils. Statistical analysis: Marissa Raskin, Marie‐H. Monfils, Jason Shumake. Obtained funding: Marie‐H. Monfils, Jasper Smits, Michael Telch, Michael Otto, Hongjoo Lee, Marissa Raskin. Study supervision: Marie‐H. MonfilsAll the authors have made substantial contributions to the design of the work, the analysis and interpretation of the data, and the drafting of the work or its critical revision.

## Funding

This study was supported by the National Institute on Alcohol Abuse and Alcoholism (R01‐AA029386, T32‐AA007471, F31‐AA030936) and the National Institute of Mental Health (R01‐MH125951, R01‐MH125949).

## Conflicts of Interest

JAJS has received grants from the National Institutes of Health, the Department of Defence and the Trauma Research and Combat Casualty Care Collaborative Prevention. He has received personal fees from Big Health, Boston University and Brown University for consulting and from Elsevier and the American Psychological Association for editorial activities. Dr. Smits also has an equity interest in Earkick and has received royalty payments from various publishers. The terms of these arrangements have been reviewed and approved by the University of Texas at Austin in accordance with its conflicts of interest policies. MWO receives compensation as an advisor to Big Health and receives or has recently received grant support from the National Institute of Mental Health, the National Institute on Drug Abuse and Big Health, United States, and he receives royalties from book publications from various publishers. MR, MO, KAS, RC, MJT, JS, RG, HJL and MHM report no conflicts of interest.

## Supporting information


**Table S1:** c‐Fos and orexin cell counts, shown as mean (SD).
**Table S2:** Significant effects from 2 × 2 × 2 ANOVAs run on each subcomponent of CO2 reactivity.
**Figure S1:** Behavioural subcomponents of CO_2_ reactivity that had significant t tests within either extinction group. During induction, among rats that received extinction and air, males displayed higher ambulation and laboured breathing than females. Among female rats in the air group, rats that received retrieval‐extinction displayed higher laboured breathing during induction than rats that received extinction. Among rats in the vapour group, laboured breathing in males was higher in the extinction group but lower in the retrieval‐extinction group than in females.
Figure S2: Relationships of the CO_2_ reactivity subcomponents that were significantly predictive of LTM with orexin/c‐Fos colocalization. The relationship of rearing during flush out 2 with orexin/c‐Fos colocalization was not significant in the extinction group (**A**) nor the retrieval‐extinction group (**B**). The relationship of ambulation during 25% hold was not significant in the extinction group (**C**) nor the retrieval‐extinction group (**D**). The relationship of ambulation during induction with orexin/c‐Fos colocalization was not significant in the extinction group (**E**) nor the retrieval‐extinction group (**F**).
Figure S3: Relationships of the three measures of LTM with orexin/c‐Fos colocalization. The relationship of sipper licks with orexin/c‐Fos colocalization was not significant in the extinction group (**A**) nor the retrieval‐extinction group (**B**). The relationship of approach during the light was not significant in the extinction group (**C**) nor the retrieval‐extinction group (**D**). The relationship of approach during the light + sipper with orexin/c‐Fos colocalization was not significant in the extinction group (**E**) nor the retrieval‐extinction group (**F**).

## Data Availability

The data that support the findings of this study are openly available at https://dataverse.tdl.org/dataverse/MonfilsFearMemoryLab.
