# Supplementary material for: CO2 Reactivity but Not CO2‐Induced Orexin/c‐Fos Colocalization Differentially Predicts Alcohol‐Seeking Behaviour After Extinction and Retrieval‐Extinction in Rats
Source: Addict Biol. 2026 Jan 22;31(1):e70116. doi: 10.1111/adb.70116 (PMC12824593; doi:10.1111/adb.70116)
Supplement: Supplementary file 1 — Table S1: c‐Fos and orexin cell counts, shown as mean (SD). Table S2: Significant effects from 2 × 2 × 2 ANOVAs run on each subcomponent of CO2 reactivity. Figure S1: Behavioural subcomponents of CO2 reactivity that had significant t tests within either extinction group. During induction, among rats that received extinction and air, males displayed higher ambulation and laboured breathing than females. Among female rats in the air group, rats that received retrieval‐extinction displayed higher laboured breathing during induction than rats that received extinction. Among rats in the vapour group, laboured breathing in males was higher in the extinction group but lower in the retrieval‐extinction group than in females. Figure S2: Relationships of the CO2 reactivity subcomponents that were significantly predictive of LTM with orexin/c‐Fos colocalization. The relationship of rearing during flush out 2 with orexin/c‐Fos colocalization was not significant in the extinction group (A) nor the retrieval‐extinction group (B). The relationship of ambulation during 25% hold was not significant in the extinction group (C) nor the retrieval‐extinction group (D). The relationship of ambulation during induction with orexin/c‐Fos colocalization was not significant in the extinction group (E) nor the retrieval‐extinction group (F). Figure S3: Relationships of the three measures of LTM with orexin/c‐Fos colocalization. The relationship of sipper licks with orexin/c‐Fos colocalization was not significant in the extinction group (A) nor the retrieval‐extinction group (B). The relationship of approach during the light was not significant in the extinction group (C) nor the retrieval‐extinction group (D). The relationship of approach during the light + sipper with orexin/c‐Fos colocalization was not significant in the extinction group (E) nor the retrieval‐extinction group (F). [file ADB-31-e70116-s001.docx]

Supporting Information Table 1: c-Fos and orexin cell counts, shown as mean (SD).

| Sex | Extinction/Retrieval | Air/Vapor | ORX | c-Fos | Co-labeled |
| --- | --- | --- | --- | --- | --- |
| F | Extinction | Air | 18.65 (8.53) | 50.89 (31.46) | 6.93 (5.92) |
|  |  | Vapor | 30.73 (9.92) | 58.03 (25.56) | 12.45 (5.58) |
|  | Retrieval-Extinction | Air | 31.25 (9.29) | 68.29 (20.61) | 14.60 (4.67) |
|  |  | Vapor | 30.53 (13.44) | 67.22 (24.56) | 12.23 (6.30) |
| M | Extinction | Air | 28.76 (10.96) | 56.60 (20.40) | 14.45 (7.63) |
|  |  | Vapor | 34.85 (13.34) | 59.66 (22.10) | 17.34 (6.70) |
|  | Retrieval-Extinction | Air | 40.40 (12.60) | 58.32 (25.95) | 15.06 (3.83) |
|  |  | Vapor | 53.29 (16.81) | 54.29 (30.10) | 15.38 (8.54) |

Supporting Information Table 2: Significant effects from 2x2x2 ANOVAs run on each subcomponent of CO_2_ reactivity.

| Effect | Behavior, phase | F-statistic(1,66) | p-value |
| --- | --- | --- | --- |
| Sex × air × extinction | Rearing during induction | 4.74 | .033 |
|  | Labored breathing during induction | 13.47 | < .001 |
| Sex × air | Rearing during flush out 2 | 4.94 | .03 |
| Sex × extinction | Ambulation during induction | 4.22 | .044 |
| Sex | Rearing during flush out 1 | 6.06 | .016 |
|  | Labored breathing during flush out 2 | 28.22 | < .001 |
| Air | Ambulation during flush out 2 | 4.83 | .031 |
|  | Labored breathing during induction | 5.51 | .022 |
|  | Grooming during flush out 2 | 4.88 | .031 |
| Extinction | Ambulation during flush out 2 | 4.81 | .032 |
|  | Grooming during flush out 2 | 6.33 | .014 |

**Significant post-hoc t-tests in CO_2_ reactivity among/between extinction groups**

Among the rats in the extinction group that received air, male rats had higher ambulation during induction than female rats (t(15.99) = 2.42, *p* = .028). During induction, labored breathing was higher: in the retrieval-extinction group than the extinction group among female rats in the vapor group (t(18.16) = 2.75, *p* = .013), in males than females among rats in the vapor group that received extinction (t(7.72) = 2.48, *p* = .040), and in females than males among rats in the vapor group that received retrieval-extinction (t(14.66) = 2.45, *p* = .028).


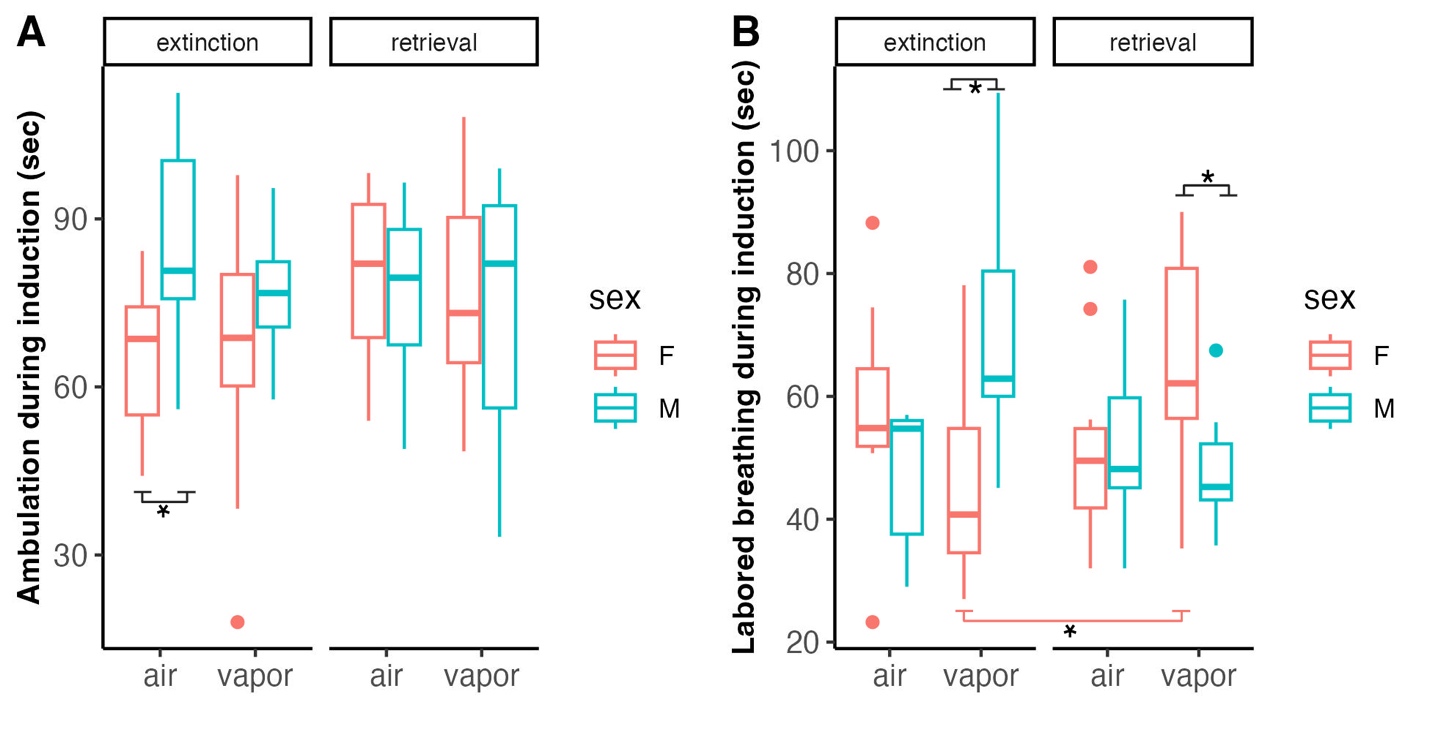
Supporting Information Figure 1: Behavioral subcomponents of CO_2_ reactivity that had significant t-tests within either extinction group. During induction, among rats that received extinction and air, males displayed higher ambulation and labored breathing than females. Among female rats in the air group, rats that received retrieval-extinction displayed higher labored breathing during induction than rats that received extinction. Among rats in the vapor group, labored breathing in males was higher in the extinction group but lower in the retrieval-extinction group than in females.

**
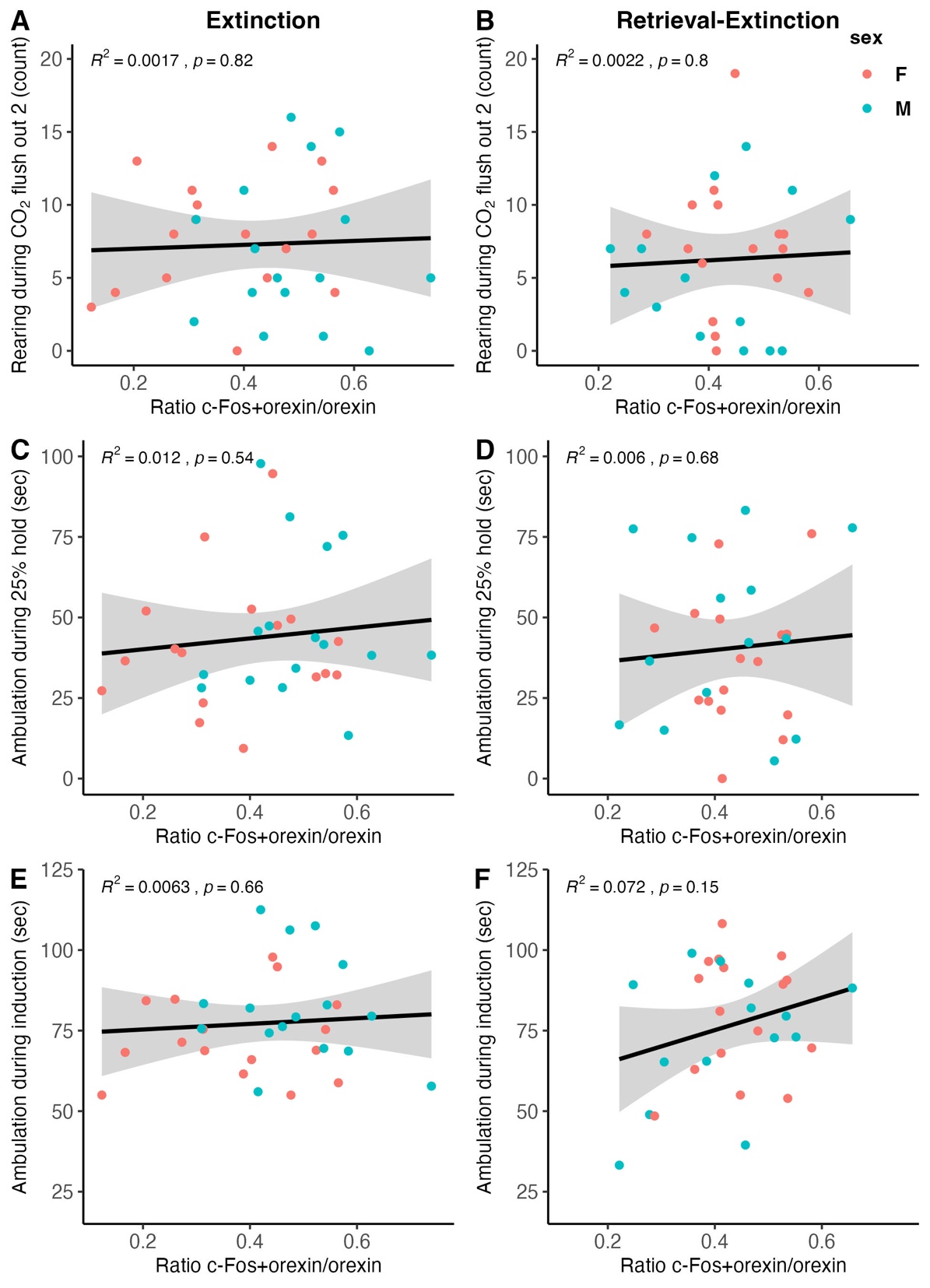
No relationship between orexin/c-Fos colocalization and CO_2_ reactivity or LTM.**

Supporting Information Figure 2: Relationships of the CO_2_ reactivity subcomponents that were significantly predictive of LTM with orexin/c-Fos colocalization. The relationship of rearing during flush out 2 with orexin/c-Fos colocalization was not significant in the extinction group **A**) nor the retrieval-extinction group **B**). The relationship of ambulation during 25% hold was not significant in the extinction group **C**) nor the retrieval-extinction group **D**). The relationship of ambulation during induction with orexin/c-Fos colocalization was not significant in the extinction group **E**) nor the retrieval-extinction group **F**).


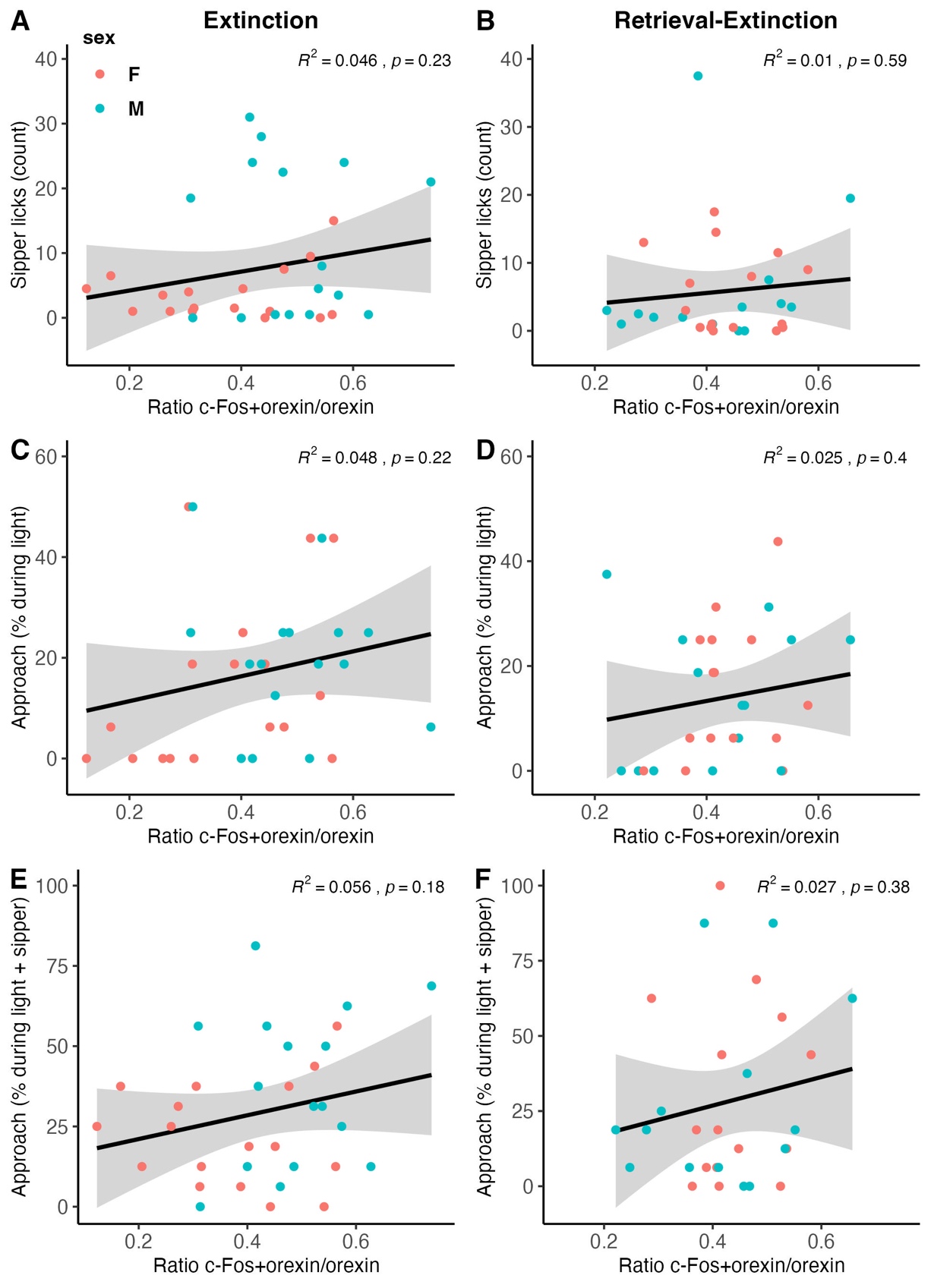


Supporting Information Figure 3: Relationships of the three measures of LTM with orexin/c-Fos colocalization. The relationship of sipper licks with orexin/c-Fos colocalization was not significant in the extinction group **A**) nor the retrieval-extinction group **B**). The relationship of approach during the light was not significant in the extinction group **C**) nor the retrieval-extinction group **D**). The relationship of approach during the light + sipper with orexin/c-Fos colocalization was not significant in the extinction group **E**) nor the retrieval-extinction group **F**).
